# Acute Subcutaneous Gastric Perforation at a Previous Percutaneous Endoscopic Gastrostomy (PEG) Site

**DOI:** 10.7759/cureus.103799

**Published:** 2026-02-17

**Authors:** Yee Wen Tan, Christopher Leung, Janaka Balasooriya

**Affiliations:** 1 General Surgery, Canberra Hospital, Australian Capital Territory (ACT) Health, Canberra, AUS

**Keywords:** abdominal sepsis, emergency exploratory laparotomy, large anterior gastric perforation, percutaneous endoscopic gastro-jejunostomy, subcutaneous abscess

## Abstract

Percutaneous endoscopic gastrostomy (PEG) is commonly used to provide long-term enteral nutrition and is generally associated with a low rate of major complications. Persistent gastrocutaneous fistula after PEG removal occurs in approximately 0.5-3.9% of cases. We report an unusual case of delayed subcutaneous gastric perforation into the subcutaneous space presenting as abdominal wall sepsis six years after PEG removal without prior history of PEG-related complications.

A 24-year-old man with a history of repaired congenital tracheoesophageal fistula and long-term PEG dependence presented with fever, abdominal pain, and rapidly progressive swelling of the left lower abdominal wall. Computed tomography demonstrated extensive subcutaneous emphysema and fluid collections concerning for necrotizing soft-tissue infection. Emergency surgical exploration revealed gastric contents tracking through the abdominal wall fascia into the subcutaneous plane at the site of the previous PEG with intact skin. A defect in the anterior gastric wall was repaired primarily with omental patching, and the fascial tract was excised and repaired. The subcutaneous collection was managed with extensive washout and drainage. The patient required intensive care for septic shock but made a good recovery following definitive surgical source control and antimicrobial therapy.

PEG site healing is usually uncomplicated, with most of the tracts closing spontaneously. True delayed reactivation after years of apparent healing is exceedingly rare. Isolated reports describe delayed enterocutaneous and gastrocolocutaneous fistulae presenting after prolonged asymptomatic periods. A previous case report described delayed subcutaneous leakage occurring two years after division of a gastrostomy tract; however, to our knowledge, spontaneous subcutaneous gastric perforation occurring six years after PEG removal, in the absence of any intervening procedure, has not previously been reported. Proposed mechanisms include persistent epithelialization of the gastrostomy tract, dense adhesions between the stomach and abdominal wall, localized ischemia, and micro-perforation related to infection. These changes may allow delayed breakdown and direct subcutaneous leakage. Radiologically, this presentation may mimic necrotizing soft-tissue infection, making early surgical exploration essential for diagnosis and source control.

Management depends on clinical severity. Endoscopic techniques such as argon plasma coagulation, clip placement, and suturing may be used in stable patients with gastrocutaneous fistulae, but in the presence of perforation, sepsis, or extensive soft-tissue contamination, surgical excision of the fistulous tract with primary gastric repair and wide drainage remains definitive. As no formal guidelines exist for this rare complication, treatment should follow general surgical principles of prompt source control, antimicrobial therapy, and supportive care, as demonstrated in this case.

This case highlights a rare but life-threatening delayed complication of PEG removal. Clinicians should maintain a high index of suspicion for late PEG-related fistulae in patients presenting with unexplained abdominal wall sepsis, even many years after gastrostomy removal, as early recognition and prompt surgical management are essential for favourable outcomes.

## Introduction

Percutaneous endoscopic gastrostomy (PEG) provides a reliable route for long-term enteral nutrition in patients unable to maintain adequate oral intake. Although generally safe, PEG placement and removal are associated with both early and late complications, ranging from minor peristomal leakage to major visceral injury and sepsis [1]. The majority of PEG tracts close spontaneously within days after tube removal [2]. However, persistent or reactivated gastrocutaneous fistulae are reported in up to 3.9% of cases and may present as chronic drainage or, rarely, acute abdominal wall sepsis [3-5].

Delayed reactivation of a PEG tract several years after removal is extremely uncommon, with only isolated case reports describing fistula formation or leakage occurring after prolonged asymptomatic intervals [6-8]. Proposed mechanisms include chronic epithelialization of the gastrostomy tract, dense adhesions between the stomach and anterior abdominal wall, and localized ischemia or infection [9]. These presentations may mimic necrotizing soft-tissue infection or abdominal wall abscess and are challenging to diagnose without any clinical suspicion.

We report the case of a young adult with a history of congenital tracheoesophageal fistula and prior long-term PEG dependence who presented with abdominal wall sepsis secondary to a delayed gastric leak through a previous gastrostomy site, several years after tube removal.

## Case presentation

A 24-year-old man presented with a two-day history of flu-like symptoms, left lower abdominal pain, nausea, vomiting, diarrhoea, lethargy, and fever. His medical history included mild intellectual disability, congenital renal dysplasia with stage 3 chronic kidney disease, a ventricular septal defect, and a repaired congenital tracheoesophageal fistula. He underwent Nissen fundoplication in infancy and was dependent on a feeding gastrostomy tube until 18 years of age. His PEG tube was subsequently removed, with no ongoing feeding issues. Following PEG removal, the wound demonstrated satisfactory healing, with no associated intermittent discharge, recent trauma, or requirement for further intervention.

**Figure 1:**
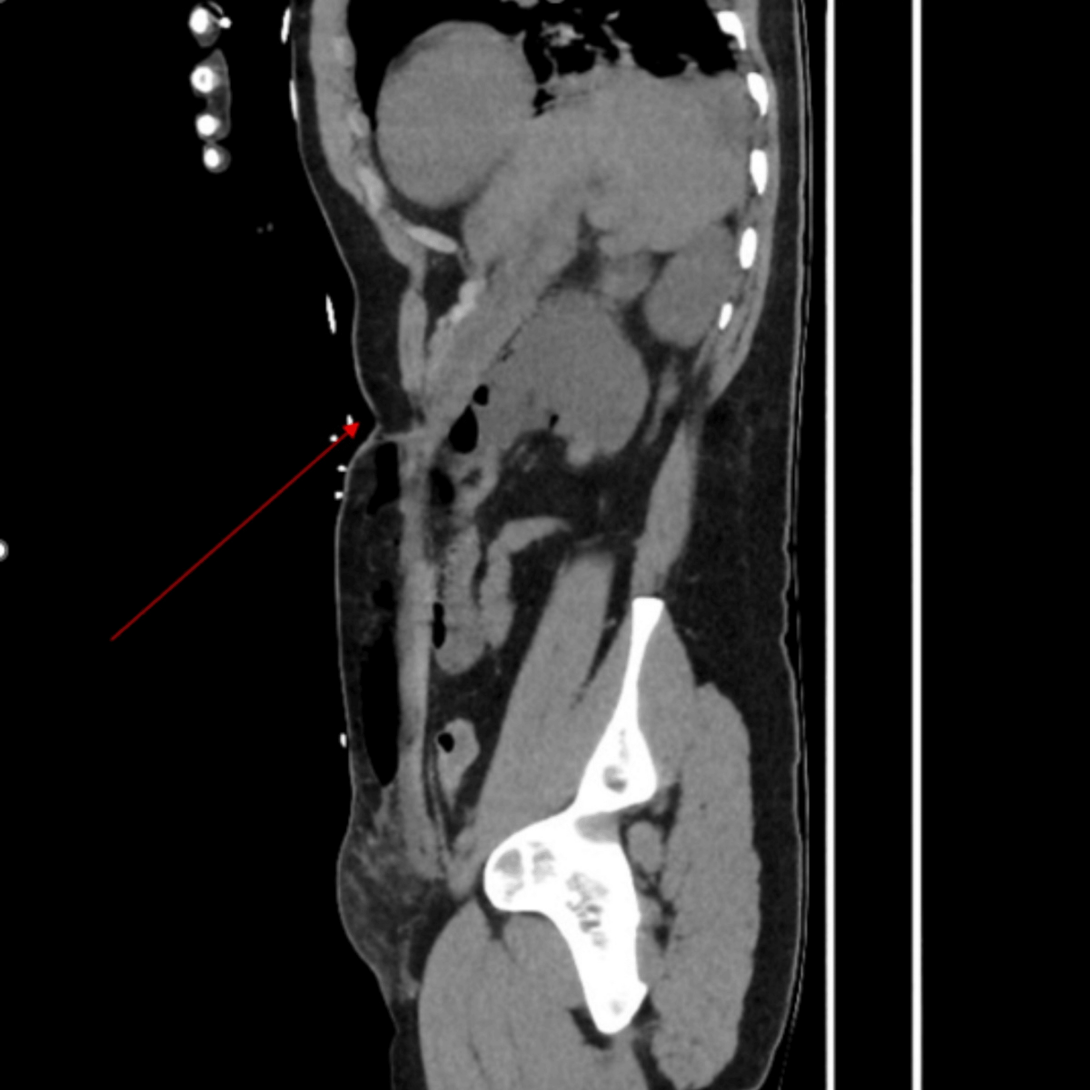
Sagittal view of computed tomography (CT) abdomen and pelvis demonstrating previous percutaneous endoscopic gastrostomy (PEG) insertion site (highlighted by a red arrow) and perforation of the stomach into the subcutaneous space, evident by gas locules within the subcutaneous layer above the anterior abdominal wall.

**Figure 2:**
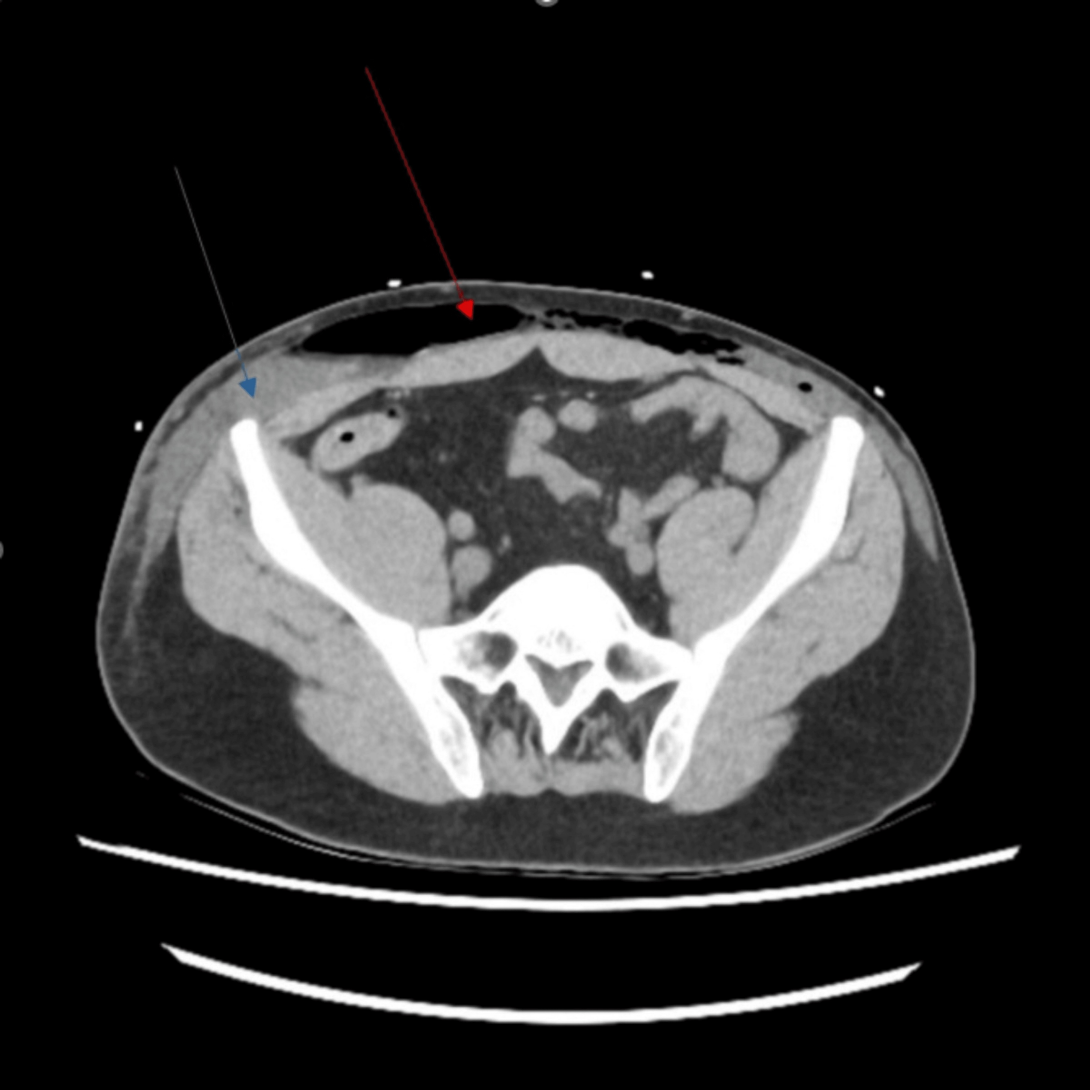
Axial view of CT abdomen demonstrating subcutaneous collection (highlighted by a blue arrow) and emphysema (highlighted by a red arrow) tracking down to the lower anterior abdominal wall and bilateral flanks, raising initial suspicion of necrotizing fasciitis.

On presentation, he was hypotensive, tachycardic, and tachypnoeic, with a distended abdomen and a tender, swollen left lower abdominal wall associated with overlying skin discolouration and crepitus extending to the left thigh. The previous PEG site was well healed. Laboratory investigations demonstrated white cell count of 4 × 10⁹/L (4.3-10.8 × 10⁹/L), C-reactive protein of 431 mg/L (<5.0 mg/L), and deranged renal function with estimated glomerular filtration rate (eGFR) of 29 mL/min/1.73 m² (> 90 mL/min/1.73 m²). Computed tomography of the abdomen and pelvis demonstrated extensive subcutaneous emphysema and fluid collections involving the anterior abdominal wall and left flank, raising concern for a necrotising soft-tissue infection (Figures 1-2).

**Figure 3:**
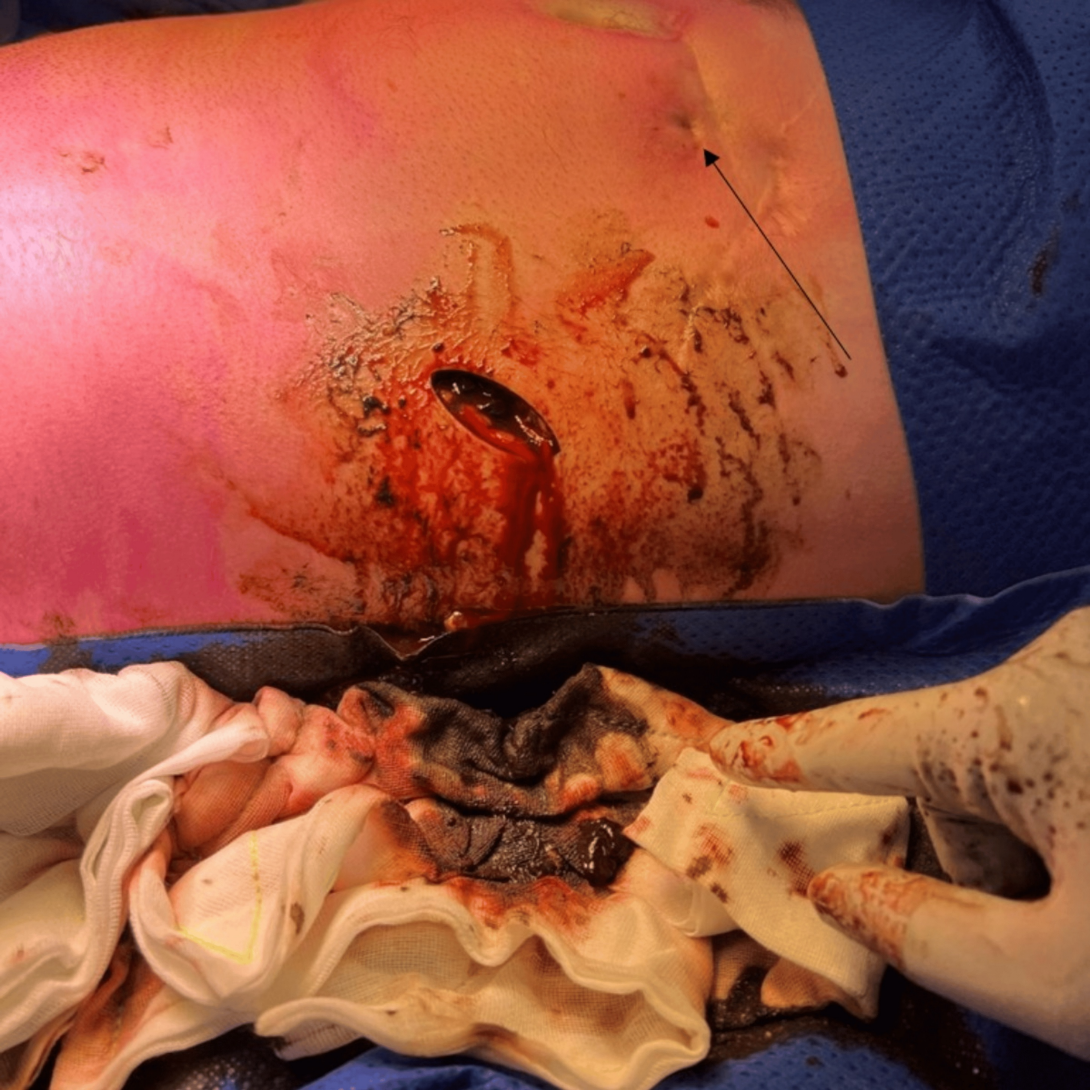
Initial incision over the left lateral lower abdominal wall that revealed gastric content tracking along the lateral abdominal wall superiorly. The previous percutaneous endoscopic gastrostomy (PEG) site was highlighted by a black arrow.

**Figure 4:**
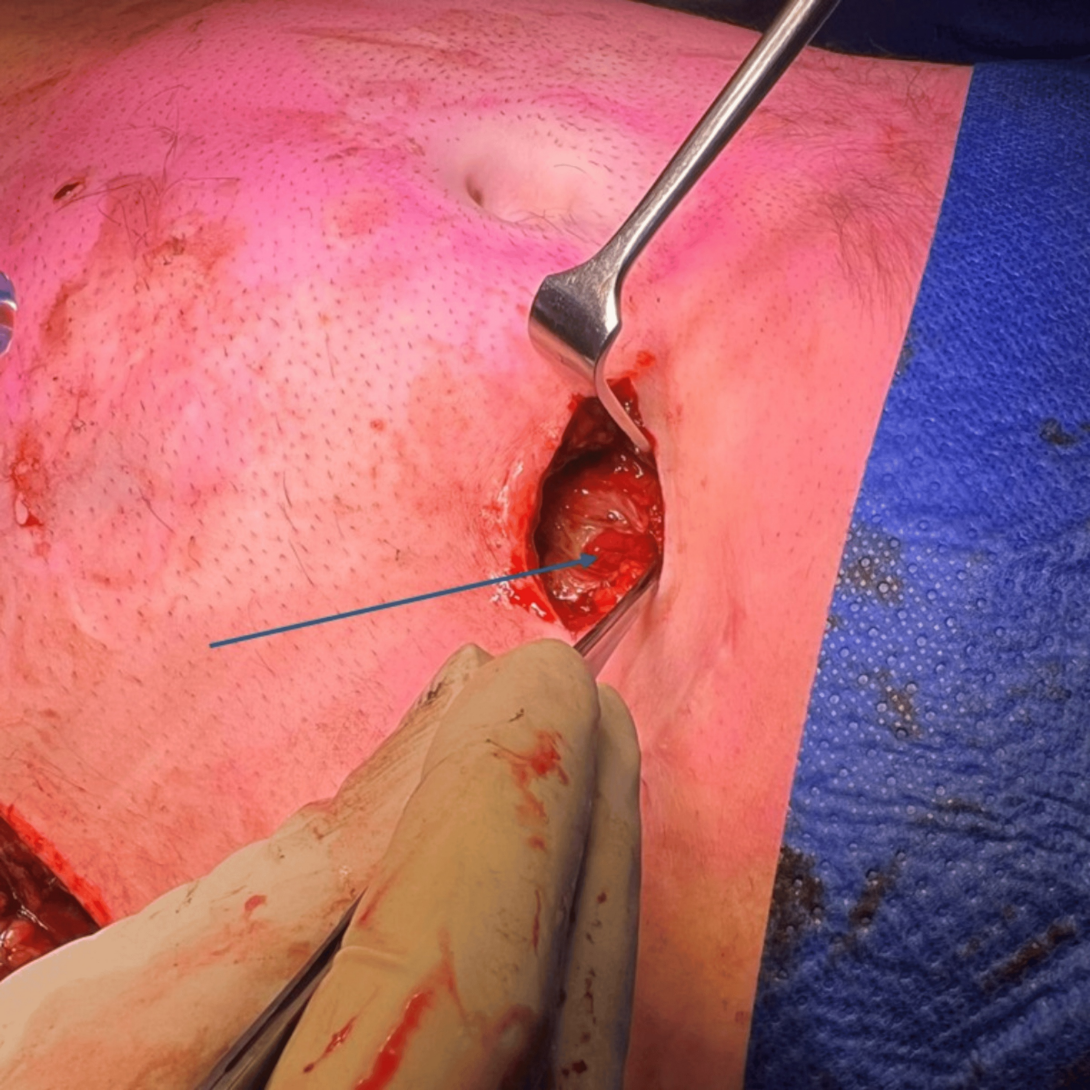
Further exploration of the previous percutaneous endoscopic gastrostomy (PEG) insertion site revealed gastric perforation (highlighted by a blue arrow) connecting to the left abdominal wall fascia.

The patient was taken urgently to the theatre for exploration. An initial incision over the most fluctuant area of the lateral abdominal wall revealed bilious gastric contents tracking along the abdominal wall (Figure 3). Further exploration at the previous PEG site identified a fascial defect communicating with the stomach, with ongoing leakage of gastric contents (Figure 4). No foreign body was identified. Due to significant inflammatory changes surrounding the fascial defect, a decision was made to proceed directly with midline laparotomy and adhesiolysis to mobilise the stomach from the anterior abdominal wall. The anterior gastric wall defect was repaired primarily in two layers, with an omental patch positioned over the repair site. The fistulous tract within the fascia was excised, and the fascial defect was closed. Extensive washout and drainage of the abdominal wall were performed, with two additional incisions and placement of subcutaneous drains.

**Figure 5:**
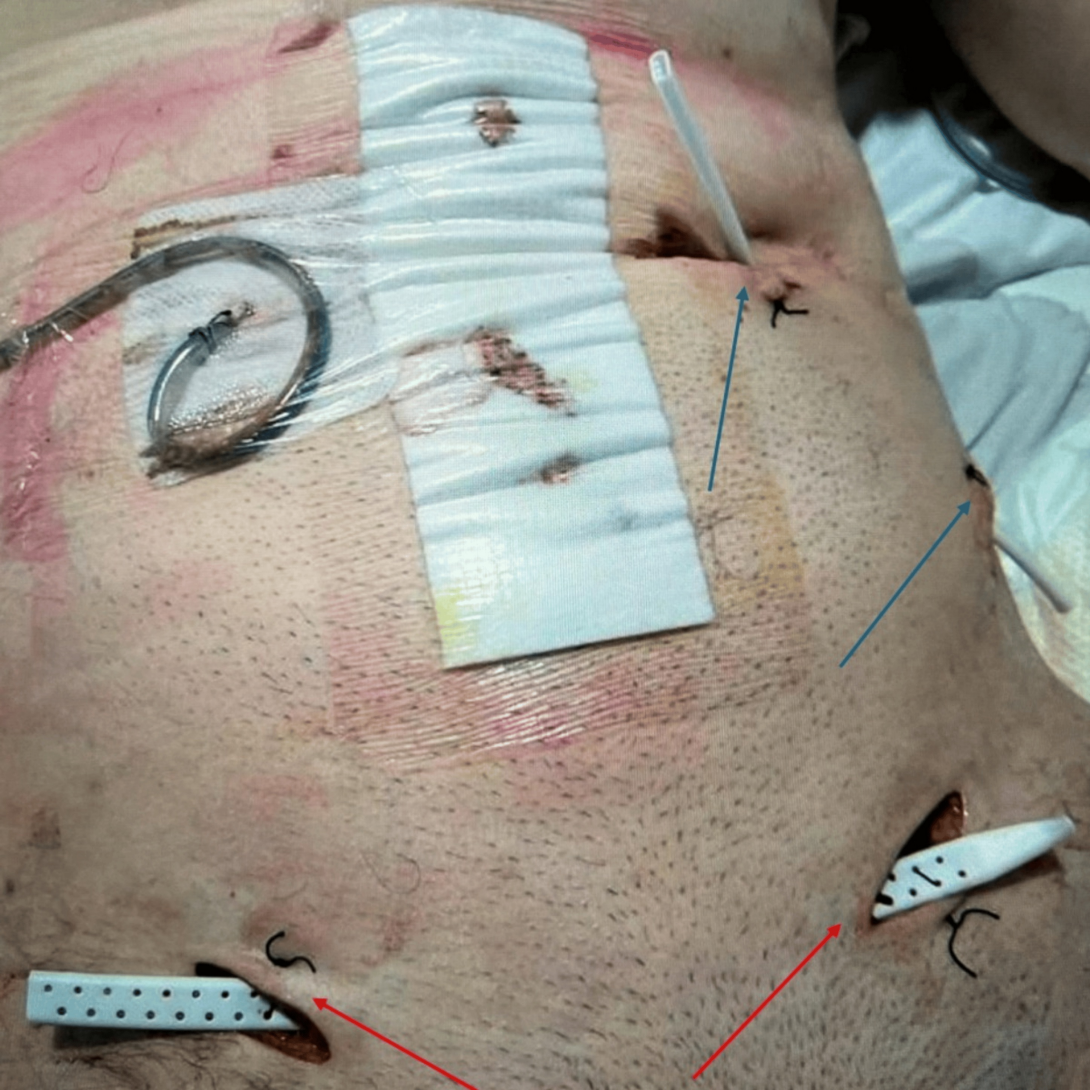
Postoperative image of the incisional wounds over the bilateral lower abdominal wall connecting to each other with a Jackson-Pratt drain within (highlighted by red arrows). Another two incisions were made over the left upper lateral abdominal wall to drain the anterior abdominal wall collection that tracts from the previous percutaneous endoscopic gastrostomy (PEG) insertion site (highlighted by blue arrows).

Postoperatively, he was admitted to the intensive care unit for monitoring and management of his septic shock and acute kidney injury. He received three days of intravenous meropenem, vancomycin, clindamycin, and fluconazole, which was then stepped down to intravenous piperacillin-tazobactam for another seven days for hospital-acquired pneumonia. Fortunately, he made a good recovery following adequate source control and adequate antibiotics and was transferred out of the ICU after three days to the surgical ward. Oral intake was gradually reintroduced, and he was discharged home in a stable condition with abdominal wall drains in situ after 12 days of hospitalization. His abdominal drains were subsequently removed once output was minimal (Figure 5). An outpatient endoscopic examination was arranged.

## Discussion

PEG is widely used for long-term enteral nutrition and is generally associated with a low rate of major complications. Early complications are well recognised, whereas late complications are less common and often present in an atypical or delayed manner [3,4]. Persistent gastrocutaneous fistula following PEG removal has been reported in approximately 0.5-3.9% of cases, with most tracts closing spontaneously within days [5]. Reactivation or delayed presentation of a fistula several years after tube removal is exceedingly rare.

Several case reports have described similar delayed presentations, including an enterocutaneous fistula diagnosed eight years after PEG insertion and gastrocolocutaneous fistulae with prolonged asymptomatic periods [6,7]. These cases highlight that PEG-related fistulae may remain clinically silent for years before re-presenting, often in the setting of infection or physiological stress. Persistent gastrocutaneous fistula is uncommon in adults, and even less common is recanalization of a fistula track after initial closure. DeSoucy et al. reported a case of delayed leakage of gastric contents into the subcutaneous tissues two years after division of a gastrostomy tract during abdominoplasty; however, spontaneous perforation into the subcutaneous plane occurring six years after PEG removal, without any intervening procedure, has not been previously reported in the literature [8]. In this case, the patient’s preceding viral illness with frequent coughing accompanied by repeated vomiting may have caused an acute increase in intragastric pressure, precipitating perforation at the vulnerable gastric wall over the previous PEG site.

Proposed mechanisms include chronic epithelialisation of the gastrostomy tract, traction from dense adhesions, buried bumper-related ischaemia, and micro-perforation associated with localised infection [9]. Subcutaneous gastric perforation is an acute breach of the gastric wall, presenting with pain, soft tissue swelling, and systemic inflammatory features, with imaging showing extraluminal air or fluid without a mature tract. In contrast, a chronic gastrocutaneous fistula is a persistent, epithelialised tract after PEG removal, usually causing controlled external leakage with minimal systemic symptoms. Radiologically, fistulae appear as well-formed tracts, whereas perforation shows tissue disruption, tissue stranding to suggest inflammation, and subcutaneous collection or free gas [10]. Delayed PEG-related complications may mimic necrotising soft tissue infection, warranting early surgical exploration for definitive diagnosis and source control.

In our case, CT imaging demonstrated direct continuity between the anterior gastric wall and a fascial defect at the previous PEG site, with extensive subcutaneous gas and fluid tracking along the abdominal wall, most conspicuous on sagittal reconstruction. These findings were highly suggestive of direct gastric perforation into the subcutaneous space. Prompt recognition of these findings is essential to prevent progression to severe soft tissue infection or sepsis. A variety of management strategies for gastrocutaneous fistulae have been described, depending on clinical severity. Minimally invasive approaches, including argon plasma coagulation, over-the-scope clip placement, and endoscopic or percutaneous suturing, have been reported in selected cases [11-13]. However, in patients presenting with sepsis or extensive soft-tissue involvement, surgical excision of the fistulous tract with primary gastric repair remains the definitive treatment.

Due to the rarity of subcutaneous gastric perforation as a late PEG complication, no formal management guidelines currently exist. In accordance with general surgical principles, prompt source control through repair of the gastric wall defect, drainage of subcutaneous collections, appropriate antimicrobial therapy, and supportive care is warranted, as demonstrated successfully in our case. Alternative approaches to gastric defect repair, such as primary mass closure incorporating the fascial defect or direct anterior gastric wall repair via fascial incision, can be considered. These options were not pursued due to extensive surrounding inflammatory changes and the associated risk of recurrence in our case.

## Conclusions

Delayed leakage from a previous PEG site is a rare but potentially life-threatening complication that may occur years after tube removal. Our case demonstrates that a healed PEG tract can remain a potential site of vulnerability for life-threatening complications years later, even in the absence of previous delayed PEG healing or gastrocutaneous fistula. Hence, clinicians should maintain a high index of suspicion in patients with prior PEG placement presenting with abdominal wall sepsis. Early imaging, prompt surgical exploration when indicated, and definitive source control are essential for favourable outcomes.
